# Bis{(*E*)-2-meth­oxy-6-[(4-methyl­phen­yl)­imino­meth­yl]phenolato}zinc(II)

**DOI:** 10.1107/S1600536808033102

**Published:** 2008-10-18

**Authors:** Hui-Duo Xian, Jian-Feng Liu, Hua-Qiong Li, Guo-Liang Zhao

**Affiliations:** a Zhejiang Key Laboratory for Reactive Chemistry on Solid Surfaces, Institute of Physical Chemistry, Zhejiang Normal University, Jinhua, Zhejiang 321004, People’s Republic of China, and, College of Chemistry and Life Science, Zhejiang Normal University, Jinhua, Zhejiang 321004, People’s Republic of China

## Abstract

The title compound, [Zn(C_15_H_14_NO_2_)_2_], contains a four-coordinate Zn atom located on a twofold rotation axis that exhibits a distorted tetra­hedral geometry by two phenolate O atoms and two azomethine N atoms of the Schiff base 2-methoxy-6-[(4-methyl­phen­yl)imino­meth­yl]phenolate ligands.

## Related literature

For related literature, see: Bhattacharyya *et al.* (2002 ▶); Iyere *et al.* (2004 ▶); Müller *et al.* (2001 ▶); Yu *et al.* (2007 ▶); Zhou & Zhao (2007 ▶).
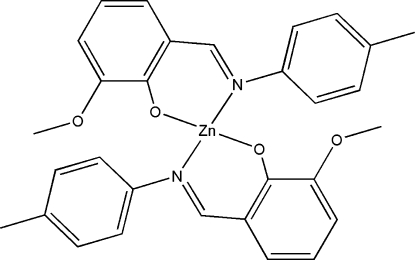


## Experimental

### 

#### Crystal data

[Zn(C_15_H_14_NO_2_)_2_]
*M* *_r_* = 545.93
Monoclinic, 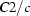

*a* = 14.0698 (4) Å
*b* = 16.3828 (5) Å
*c* = 12.0532 (3) Å
β = 107.5880 (10)°
*V* = 2648.42 (13) Å^3^
*Z* = 4
Mo *K*α radiation
μ = 0.97 mm^−1^
*T* = 296 (2) K
0.52 × 0.08 × 0.08 mm

#### Data collection

Bruker APEXII area-detector diffractometer
Absorption correction: multi-scan (*SADABS*; Sheldrick, 1996 ▶) *T*_min_ = 0.914, *T*_max_ = 0.930
10967 measured reflections
3013 independent reflections
2523 reflections with *I* > 2σ(*I*)
*R*_int_ = 0.025

#### Refinement

*R*[*F* ^2^ > 2σ(*F* ^2^)] = 0.033
*wR*(*F* ^2^) = 0.096
*S* = 1.03
3013 reflections
168 parameters
H-atom parameters constrained
Δρ_max_ = 0.35 e Å^−3^
Δρ_min_ = −0.27 e Å^−3^

### 

Data collection: *APEX2* (Bruker, 2006 ▶); cell refinement: *SAINT* (Bruker, 2006 ▶); data reduction: *SAINT*; program(s) used to solve structure: *SHELXS97* (Sheldrick, 2008 ▶); program(s) used to refine structure: *SHELXL97* (Sheldrick, 2008 ▶); molecular graphics: *SHELXTL* (Sheldrick, 2008 ▶); software used to prepare material for publication: *SHELXL97*.

## Supplementary Material

Crystal structure: contains datablocks I, global. DOI: 10.1107/S1600536808033102/at2636sup1.cif

Structure factors: contains datablocks I. DOI: 10.1107/S1600536808033102/at2636Isup2.hkl

Additional supplementary materials:  crystallographic information; 3D view; checkCIF report

## Figures and Tables

**Fig. 1.**
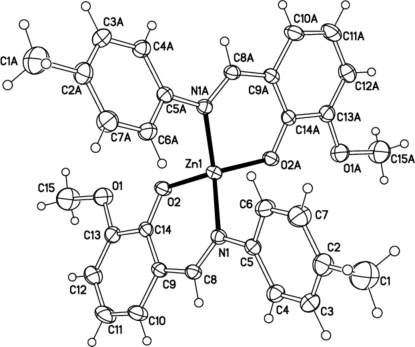
The molecular structure of (I), showing the atom-labelling scheme. Displacement ellipsoids are drawn at the 30% probability level.

## supplementary crystallographic information

### Comment

Schiff base ligands derived from substituted salicylaldehyde and aniline and their metal complexes have been widely investigated because of their novel structural features (Müller *et al.*, 2001; Bhattacharyya *et al.*, 2002). They include complexes with a methoxy group in the *ortho* position as the methoxy group can also bind to the metal. Such Schiff bases behave as bidentate ligands to divalent first-row transition metals (Zhou & Zhao, 2007). Similar cobalt (II) complexes have been reported by Iyere *et al.* (2004). Here, we describe the synthesis and crystal structure of a zinc complex, (I), of a Schiff base derived from *o*-vanillin and *p*-toluidine.

The structural features of the (I) dimer shown in Fig.ure 1. The Zn atom sits on a twofold axis. The tridentate ligands coordinate to the Zinc ion through the phenolic hydroxy O atom and the azomethine N atom, forming a distorted tetrahedral geometry around the metal ion. It is different from the complex [Zn*L*_2_(NO_3_)_2_] (Yu *et al.*, 2007) in which Zn is coordinated by the methoxy O atom and the azomethine N atom.

### Experimental

The ligand was prepared by the direct solid-phase reaction of *o*-vanillin (10 mmol, 1.5251 g) and *p*-toluidine (10 mmol, 1.0700 g). The reactants were ground in an agate mortar. The colour of the mixture changed from light yellow to orange. A solution of Zn(C_2_O_4_) (1 mmol, 0.153 g) in methanol (10 ml) was added to a methanol solution of the Schiff base ligand (2 mmol, 0.48 g). orange crystals were isolated after two weeks.

### Refinement

The H atoms bonded to C atoms were positioned geometrically and refined using a riding model [aromatic C—H = 0.93 Å, aliphatic C—H = 0.96 Å, aliphatic C—H = 0.97 Å, and *U*_iso_(H) = 1.2*U*_eq_(C)].

### Crystal data

**Table:**

[Zn(C_15_H_14_NO_2_)_2_]	*F*(000) = 1136
*M**_r_* = 545.93	*D*_x_ = 1.369 Mg m^−^^3^
Monoclinic, *C*2/*c*	Mo *K*α radiation, λ = 0.71073 Å
Hall symbol: -C 2yc	Cell parameters from 4200 reflections
*a* = 14.0698 (4) Å	θ = 2.0–27.5°
*b* = 16.3828 (5) Å	µ = 0.97 mm^−^^1^
*c* = 12.0532 (3) Å	*T* = 296 K
β = 107.588 (1)°	Prism, orange
*V* = 2648.42 (13) Å^3^	0.52 × 0.08 × 0.08 mm
*Z* = 4	

### Data collection

**Table:**

Bruker APEXII diffractometer	3013 independent reflections
Radiation source: fine-focus sealed tube	2523 reflections with *I* > 2σ(*I*)
graphite	*R*_int_ = 0.025
ω scans	θ_max_ = 27.5°, θ_min_ = 2.0°
Absorption correction: multi-scan (SADABS; Sheldrick, 1996)	*h* = −18→18
*T*_min_ = 0.914, *T*_max_ = 0.930	*k* = −16→21
10967 measured reflections	*l* = −15→15

### Refinement

**Table:**

Refinement on *F*^2^	Primary atom site location: structure-invariant direct methods
Least-squares matrix: full	Secondary atom site location: difference Fourier map
*R*[*F*^2^ > 2σ(*F*^2^)] = 0.033	Hydrogen site location: inferred from neighbouring sites
*wR*(*F*^2^) = 0.096	H-atom parameters constrained
*S* = 1.03	*w* = 1/[σ^2^(*F*_o_^2^) + (0.0548*P*)^2^ + 1.3911*P*] where *P* = (*F*_o_^2^ + 2*F*_c_^2^)/3
3013 reflections	(Δ/σ)_max_ < 0.001
168 parameters	Δρ_max_ = 0.35 e Å^−^^3^
0 restraints	Δρ_min_ = −0.27 e Å^−^^3^

### Special details

**Table:**

Geometry. All e.s.d.'s (except the e.s.d. in the dihedral angle between two l.s. planes) are estimated using the full covariance matrix. The cell e.s.d.'s are taken into account individually in the estimation of e.s.d.'s in distances, angles and torsion angles; correlations between e.s.d.'s in cell parameters are only used when they are defined by crystal symmetry. An approximate (isotropic) treatment of cell e.s.d.'s is used for estimating e.s.d.'s involving l.s. planes.
Refinement. Refinement of *F*^2^ against ALL reflections. The weighted *R*-factor *wR* and goodness of fit *S* are based on *F*^2^, conventional *R*-factors *R* are based on *F*, with *F* set to zero for negative *F*^2^. The threshold expression of *F*^2^ > σ(*F*^2^) is used only for calculating *R*-factors(gt) *etc*. and is not relevant to the choice of reflections for refinement. *R*-factors based on *F*^2^ are statistically about twice as large as those based on *F*, and *R*- factors based on ALL data will be even larger.

### Fractional atomic coordinates and isotropic or equivalent isotropic displacement parameters (Å^2^)

**Table:**

	*x*	*y*	*z*	*U*_iso_*/*U*_eq_	
Zn1	0.0000	0.029267 (18)	0.2500	0.03242 (12)	
N1	0.04480 (11)	0.08214 (9)	0.12438 (13)	0.0314 (3)	
O1	0.26186 (12)	−0.13463 (10)	0.42256 (14)	0.0539 (4)	
O2	0.11926 (10)	−0.03384 (8)	0.31639 (12)	0.0386 (3)	
C1	−0.1868 (3)	0.3361 (2)	−0.1614 (3)	0.0881 (10)	
H1A	−0.1785	0.3347	−0.2376	0.132*	
H1B	−0.1641	0.3876	−0.1253	0.132*	
H1C	−0.2561	0.3291	−0.1680	0.132*	
C2	−0.12681 (17)	0.26797 (15)	−0.0881 (2)	0.0526 (6)	
C3	−0.07130 (16)	0.21486 (15)	−0.13218 (19)	0.0479 (5)	
H3A	−0.0719	0.2207	−0.2091	0.058*	
C4	−0.01499 (15)	0.15341 (13)	−0.06598 (17)	0.0393 (5)	
H4A	0.0220	0.1188	−0.0982	0.047*	
C5	−0.01341 (13)	0.14317 (11)	0.04915 (16)	0.0326 (4)	
C6	−0.07168 (16)	0.19465 (15)	0.09294 (19)	0.0481 (5)	
H6A	−0.0740	0.1874	0.1686	0.058*	
C7	−0.12619 (19)	0.25644 (17)	0.0254 (2)	0.0587 (6)	
H7A	−0.1634	0.2912	0.0571	0.070*	
C8	0.12692 (13)	0.05742 (13)	0.10587 (17)	0.0344 (4)	
H8A	0.1411	0.0807	0.0423	0.041*	
C9	0.19768 (14)	−0.00060 (13)	0.17051 (17)	0.0340 (4)	
C10	0.28175 (17)	−0.01304 (15)	0.1298 (2)	0.0492 (6)	
H10A	0.2847	0.0137	0.0629	0.059*	
C11	0.35710 (17)	−0.06289 (18)	0.1865 (2)	0.0611 (7)	
H11A	0.4118	−0.0695	0.1593	0.073*	
C12	0.35297 (16)	−0.10458 (16)	0.2861 (2)	0.0541 (6)	
H12A	0.4050	−0.1390	0.3248	0.065*	
C13	0.27268 (15)	−0.09502 (13)	0.32723 (18)	0.0411 (5)	
C14	0.19242 (13)	−0.04184 (11)	0.27139 (17)	0.0321 (4)	
C15	0.3448 (2)	−0.1804 (2)	0.4906 (3)	0.0850 (10)	
H15A	0.3283	−0.2056	0.5543	0.128*	
H15B	0.4009	−0.1448	0.5202	0.128*	
H15C	0.3612	−0.2219	0.4431	0.128*	

### Atomic displacement parameters (Å^2^)

**Table:**

	*U*^11^	*U*^22^	*U*^33^	*U*^12^	*U*^13^	*U*^23^
Zn1	0.02977 (17)	0.0356 (2)	0.0350 (2)	0.000	0.01448 (13)	0.000
N1	0.0318 (7)	0.0305 (8)	0.0330 (8)	−0.0016 (6)	0.0113 (6)	−0.0010 (6)
O1	0.0533 (9)	0.0591 (10)	0.0499 (10)	0.0229 (8)	0.0166 (8)	0.0156 (8)
O2	0.0340 (7)	0.0464 (9)	0.0391 (8)	0.0076 (6)	0.0167 (6)	0.0083 (6)
C1	0.094 (2)	0.087 (2)	0.086 (2)	0.0422 (19)	0.0316 (19)	0.0423 (19)
C2	0.0495 (12)	0.0524 (14)	0.0538 (14)	0.0119 (11)	0.0124 (11)	0.0160 (11)
C3	0.0497 (12)	0.0564 (14)	0.0368 (12)	0.0018 (10)	0.0118 (10)	0.0095 (10)
C4	0.0425 (10)	0.0401 (12)	0.0357 (11)	0.0003 (9)	0.0123 (9)	−0.0037 (9)
C5	0.0322 (9)	0.0305 (10)	0.0356 (10)	−0.0026 (7)	0.0110 (8)	0.0003 (8)
C6	0.0486 (12)	0.0614 (15)	0.0368 (12)	0.0157 (11)	0.0166 (10)	0.0043 (10)
C7	0.0610 (14)	0.0629 (16)	0.0547 (14)	0.0272 (13)	0.0213 (12)	0.0037 (12)
C8	0.0344 (9)	0.0373 (10)	0.0344 (10)	−0.0048 (8)	0.0148 (8)	0.0001 (8)
C9	0.0308 (9)	0.0358 (10)	0.0378 (11)	−0.0009 (8)	0.0143 (8)	−0.0030 (9)
C10	0.0444 (12)	0.0583 (15)	0.0539 (14)	0.0054 (10)	0.0283 (11)	0.0048 (11)
C11	0.0429 (12)	0.0791 (18)	0.0708 (17)	0.0175 (13)	0.0312 (12)	0.0049 (15)
C12	0.0404 (11)	0.0606 (15)	0.0620 (15)	0.0184 (11)	0.0167 (11)	0.0040 (12)
C13	0.0399 (10)	0.0424 (12)	0.0400 (11)	0.0060 (9)	0.0106 (9)	−0.0013 (9)
C14	0.0291 (9)	0.0324 (10)	0.0349 (10)	−0.0012 (7)	0.0096 (8)	−0.0057 (8)
C15	0.078 (2)	0.098 (2)	0.077 (2)	0.0455 (18)	0.0210 (16)	0.0416 (19)

### Geometric parameters (Å, °)

**Table:**

Zn1—O2^i^	1.9270 (13)	C5—C6	1.387 (3)
Zn1—O2	1.9270 (13)	C6—C7	1.378 (3)
Zn1—N1	2.0043 (15)	C6—H6A	0.9300
Zn1—N1^i^	2.0043 (15)	C7—H7A	0.9300
N1—C8	1.306 (2)	C8—C9	1.426 (3)
N1—C5	1.430 (2)	C8—H8A	0.9300
O1—C13	1.368 (2)	C9—C14	1.412 (3)
O1—C15	1.421 (3)	C9—C10	1.426 (2)
O2—C14	1.307 (2)	C10—C11	1.349 (3)
C1—C2	1.512 (3)	C10—H10A	0.9300
C1—H1A	0.9600	C11—C12	1.398 (3)
C1—H1B	0.9600	C11—H11A	0.9300
C1—H1C	0.9600	C12—C13	1.373 (3)
C2—C3	1.378 (3)	C12—H12A	0.9300
C2—C7	1.379 (3)	C13—C14	1.424 (3)
C3—C4	1.377 (3)	C15—H15A	0.9600
C3—H3A	0.9300	C15—H15B	0.9600
C4—C5	1.391 (3)	C15—H15C	0.9600
C4—H4A	0.9300		
			
O2^i^—Zn1—O2	115.11 (9)	C5—C6—H6A	119.7
O2^i^—Zn1—N1	110.57 (6)	C6—C7—C2	121.5 (2)
O2—Zn1—N1	96.45 (6)	C6—C7—H7A	119.3
O2^i^—Zn1—N1^i^	96.45 (6)	C2—C7—H7A	119.3
O2—Zn1—N1^i^	110.57 (6)	N1—C8—C9	128.41 (17)
N1—Zn1—N1^i^	128.79 (9)	N1—C8—H8A	115.8
C8—N1—C5	118.39 (15)	C9—C8—H8A	115.8
C8—N1—Zn1	119.45 (13)	C14—C9—C10	119.53 (19)
C5—N1—Zn1	122.05 (11)	C14—C9—C8	125.43 (16)
C13—O1—C15	117.05 (19)	C10—C9—C8	114.97 (18)
C14—O2—Zn1	125.29 (12)	C11—C10—C9	121.3 (2)
C2—C1—H1A	109.5	C11—C10—H10A	119.4
C2—C1—H1B	109.5	C9—C10—H10A	119.4
H1A—C1—H1B	109.5	C10—C11—C12	120.0 (2)
C2—C1—H1C	109.5	C10—C11—H11A	120.0
H1A—C1—H1C	109.5	C12—C11—H11A	120.0
H1B—C1—H1C	109.5	C13—C12—C11	120.4 (2)
C3—C2—C7	117.5 (2)	C13—C12—H12A	119.8
C3—C2—C1	121.5 (2)	C11—C12—H12A	119.8
C7—C2—C1	121.0 (2)	O1—C13—C12	124.2 (2)
C4—C3—C2	122.1 (2)	O1—C13—C14	114.43 (16)
C4—C3—H3A	118.9	C12—C13—C14	121.4 (2)
C2—C3—H3A	118.9	O2—C14—C9	124.19 (17)
C3—C4—C5	119.94 (19)	O2—C14—C13	118.47 (17)
C3—C4—H4A	120.0	C9—C14—C13	117.33 (16)
C5—C4—H4A	120.0	O1—C15—H15A	109.5
C6—C5—C4	118.24 (19)	O1—C15—H15B	109.5
C6—C5—N1	118.37 (17)	H15A—C15—H15B	109.5
C4—C5—N1	123.39 (17)	O1—C15—H15C	109.5
C7—C6—C5	120.6 (2)	H15A—C15—H15C	109.5
C7—C6—H6A	119.7	H15B—C15—H15C	109.5
			
O2^i^—Zn1—N1—C8	−111.17 (15)	C5—N1—C8—C9	178.22 (19)
O2—Zn1—N1—C8	8.73 (15)	Zn1—N1—C8—C9	−5.4 (3)
N1^i^—Zn1—N1—C8	131.72 (15)	N1—C8—C9—C14	−1.6 (3)
O2^i^—Zn1—N1—C5	65.06 (15)	N1—C8—C9—C10	−178.4 (2)
O2—Zn1—N1—C5	−175.04 (14)	C14—C9—C10—C11	−0.7 (4)
N1^i^—Zn1—N1—C5	−52.05 (13)	C8—C9—C10—C11	176.4 (2)
O2^i^—Zn1—O2—C14	107.71 (16)	C9—C10—C11—C12	1.0 (4)
N1—Zn1—O2—C14	−8.61 (16)	C10—C11—C12—C13	−0.2 (4)
N1^i^—Zn1—O2—C14	−144.32 (15)	C15—O1—C13—C12	8.0 (4)
C7—C2—C3—C4	−1.5 (4)	C15—O1—C13—C14	−172.3 (2)
C1—C2—C3—C4	178.9 (2)	C11—C12—C13—O1	178.7 (2)
C2—C3—C4—C5	0.4 (3)	C11—C12—C13—C14	−1.0 (4)
C3—C4—C5—C6	1.7 (3)	Zn1—O2—C14—C9	4.4 (3)
C3—C4—C5—N1	−178.54 (18)	Zn1—O2—C14—C13	−175.95 (14)
C8—N1—C5—C6	−151.95 (19)	C10—C9—C14—O2	179.16 (19)
Zn1—N1—C5—C6	31.8 (2)	C8—C9—C14—O2	2.4 (3)
C8—N1—C5—C4	28.3 (3)	C10—C9—C14—C13	−0.5 (3)
Zn1—N1—C5—C4	−147.93 (15)	C8—C9—C14—C13	−177.21 (19)
C4—C5—C6—C7	−2.8 (3)	O1—C13—C14—O2	1.9 (3)
N1—C5—C6—C7	177.5 (2)	C12—C13—C14—O2	−178.3 (2)
C5—C6—C7—C2	1.7 (4)	O1—C13—C14—C9	−178.41 (18)
C3—C2—C7—C6	0.5 (4)	C12—C13—C14—C9	1.3 (3)
C1—C2—C7—C6	−179.9 (3)		

Symmetry codes: (i) −*x*, *y*, −*z*+1/2.
